# Iodine and Myo-Inositol: A Novel Promising Combination for Iodine Deficiency

**DOI:** 10.3389/fendo.2019.00457

**Published:** 2019-07-16

**Authors:** Daniele Barbaro, Beatrice Orrù, Vittorio Unfer

**Affiliations:** ^1^U.O. Endocrinologia, ASL Nord Ovest Toscana, Livorno, Italy; ^2^Medical Affairs Department, Lo.Li. Pharma, Rome, Italy; ^3^Department of Experimental Medicine, Sapienza University, Rome, Italy

**Keywords:** iodine deficiency, iodine, myo-inositol, thyroid, goiter, organification

## Abstract

Despite universal salt iodization programmes implemented over the last decades, iodine deficiency remains a major public health problem in many countries worldwide. Endeavors are still required to ensure sufficient iodine intake in the populations at risk in order to eliminate deficiency. Iodine is crucial for the synthesis of thyroid hormones triiodothyronine (T_3_) and thyroxine (T_4_), as well as for the thyroid health. When iodine levels are insufficient, T_4_ attests toward the lower limit of the physiological range, causing subtle fluctuations in the T_3_:T_4_ ratio. Monitoring these variations may be an accurate way to assess patient's iodine status. Recently, a number of published clinical studies documented a growing interest toward the use of myo-inositol in thyroid diseases. Myo-inositol, a carbocyclic polyol, regulates the generation of hydrogen peroxide (H_2_O_2_) in thyrocytes, crucial for iodine organification and thyroid hormone biosynthesis. Thus, combined supplementation of iodine and myo-inositol may promote higher iodine availability in thyrocytes improving thyroid functionality. This review presents novel strategies for the diagnosis and the management of iodine deficiency, focusing on the potential role of myo-inositol combined with iodine.

## Introduction

Iodine deficiency (ID) is a major health problem in many countries worldwide. Even though the salt iodization programme has been operational for decades, 1.9 billion people are estimated at risk for ID (1). In case of severe ID, endemic goiter occurs, leading to cretinism as its most serious manifestation. Less severe forms have a broader spectrum of clinical manifestations. A higher incidence of ID is found among preschool children and gestating women in low-income countries. The condition exposes to several risks such as stillbirth, miscarriage, poor growth, and cognitive impairment (2). Several studies reported that even mild-to-moderate ID during pregnancy may have long-term adverse effects on child cognition (3). Goiter is the earliest sign of ID disorders (4) and incidence rate exceeds higher than 20% amongst children and women (5). Since iodine is needed to produce thyroid hormones, diminished levels can lead to hypothyroidism at any stage of life (6). Hence, the primary need is to drive the global elimination of ID (7).

Until the 1990s, the main indicator for the assessment of ID was the incidence of total goiter prevalence (TGP). Nowadays, urinary iodine (UI) concentration is used as a reliable indicator of iodine nutrition in the population. Interventions are planned accordingly. Surveillance is primarily done in preschool- and school-aged children to classify the national iodine status and the UI concentration distribution (8). According to the International Council for Control of Iodine Deficiency Disorders median UI concentrations below 100 μg/L for non-pregnant women and children define iodine deficient populations, while normal values for pregnant women should be between 150 and 249 μg/L. Regional and worldwide estimates of iodine status were collected between 1993 and 2003 from 126 countries (9). Only 75 of these have nationally representative surveys reporting data on UI that cover 45.7% of the school-age children population. These surveys have estimated in 2003 a global TGP incidence of 15.8%. Universal salt iodization is the most widely used strategy to manage ID, and globally, 68% of households worldwide now have access to iodized salt (10). The iodization program was previously restricted to the areas with severe endemic ID or with no access to iodized salt (2, 11). However, the presence of severe risks correlated with ID has extended the urgency of iodine supplementation to children and pregnant women worldwide (12). The survey in 2003 estimated that the iodine intake in school-aged children worldwide is insufficient (UI < 100 μg/L), covering ~285 million children (36.5%) (9) (Table 1). Iodine intake is insufficient (UI < 100 μg/L) in 35.2% of the general population. Among the World Health Organization (WHO) regions, the European general population has the highest proportion of insufficient iodine intake (56.9%) (9) (Table 1), while only 66% of school-aged children having adequate intakes in 2015 (13). Salt iodization programme has now started in many countries worldwide, and 71% of the population is covered by iodized salt (10). As recommended by WHO a low salt intake is advisable, and it should be iodized or “fortified” with iodine. According to the WHO guidelines, 150 μg per day of iodine are recommended for adults and 90–120 μg for children. During pregnancy a higher iodine intake is recommended (250 μg per day) because of additional thyroid hormones required to cover both maternal and fetal needs (2). In fact, fetal thyroid begins to work around the 12th week of gestation and, in early pregnancy, the fetus relies exclusively on maternal thyroid hormones supplied through the placenta. Therefore, an adequate nutritional intake of iodine guarantees maternal euthyroidism and fetus health. The Italian guidelines LARN (Reference Intake of nutrients and energy for Italian Population) (14), suggest the supplementation with 220 μg of iodine per day during pregnancy and with 290 μg during breastfeeding. Nevertheless, a survey study reported inadequate iodine intakes in two thirds of pregnant women in Europe (15). In Italy, the National Observatory for Monitoring the Iodine prophylaxis (OSNAMI) carries out routine surveillance activities. An interesting topic recently highlighted by a meta-analysis is the link between different levels of iodine intake and thyroid disease. Indeed, a higher incidence of thyroid nodules was observed in areas with medium and low iodine concentrations, compared to those with higher concentrations (16). This note was largely addressed also in the Medical Guidelines of the American Association of Clinical Endocrinologists (AACE), the American College of Endocrinology (ACE), and the Associazione Medici Endocrinologi (AME), where iodine supplementation is recommended in iodine-deficient geographic regions for the treatment of nodules (6). The importance of adequate nutritional intakes of iodine lies in the fact that it is an essential constituent of the thyroid hormones, triiodothyronine (T_3_) and thyroxine (T_4_). Such hormones play a critical role in cell differentiation, in the development of the central nervous system at the early stages of life and contribute to maintain the metabolic homeostasis during adult life. Recently, a number of published clinical trials documented a growing interest toward the use of myo-inositol for restoring proper functioning of the thyroid. Since the use of myo-inositol is gaining a positive opinion in the endocrinological field, exploring its potential role in the ID management is very appealing.

**Table 1:** Percentage of population (school-age children, 6–12 years and general population, all age groups) with insufficient iodine intake (UI < 100 μg/L) by WHO region: surveys carried out in 2003^*^.

**WHO region**	**School-age children insufficient iodine intake (%)**	**General population insufficient iodine intake (%)**
Africa	42.3	42.6
Americas	10.1	9.8
South-East Asia	39.9	39.8
Europe	59.9	56.9
Eastern Mediterranean	55.4	54.1
Western Pacific	26.2	24.0
Total	36.5	35.2

* *Modified from WHO (9)*.

## Thyroid Hormones

Thyroid hormones T_3_ and T_4_ are produced and released through four important processes: uptake and oxidation of iodide, iodination of tyrosine, synthesis, resorption, and proteolysis of thyroglobulin (Tg). These processes depend on the availability of thyroid stimulating hormone (TSH) and iodine. Physiologically, a daily amount of 75 μg of iodine is required to produce 85 μg of T_4_. The thyroid actively incorporates the iodide through the basolateral plasma membrane of thyrocytes by the sodium/iodide symporter (NIS). Afterward the intracellular iodide is deposited in the lumen of thyroid follicles, storage site of Tg—a 660 kDa glycoprotein whose tyrosyl residues serve as substrate for iodination and hormone formation. The enzyme thyroid Peroxidase (TPO), sitting at the apical plasma membrane, uses hydrogen peroxide (H_2_O_2_) to oxidize and incorporate iodide in the tyrosyl groups of Tg. Dual oxidase (DUOX), a NADPH enzyme, endogenously generates H_2_O_2_ necessary for the process of the apex of the thyrocyte. Initial iodination of tyrosyl residues of Tg produces monoiodotyrosine (MIT) and diiodotyrosine (DIT). Homocoupling of DIT produces T_4_, while the coupling of DIT and MIT produces T_3_; both reactions are TPO-mediated. When thyroid hormones are required, Tg is transferred to the apical pole of thyrocytes, where it is then digested by proteases, namely endopeptidases cathepsins B, L, D, and exopeptidases. Following Tg digestion, hormones T_3_ and T_4_ are released. Approximately 70% of iodine from Tg (non-hormonal iodine) is recaptured intra-thyroidally by DEHAL1, an iodotyrosine deiodinase, and recycled within the gland. TSH is the stimulator that affects virtually every stage of thyroid hormone synthesis and release. The thyroid gland produces mainly T_4_, which reaches the organs where it is converted into T_3_, the most active form of thyroid hormones (17). The conversion of T_4_ into T_3_ is mediated by 5′-iodothyronine deiodinase either type I or II. Physiologically, a small amount of T_4_ is converted to reverse T_3_ by a 5-deiodinase (type III deiodinase): 80% of circulating T_3_ derives from deiodinase type I in liver and kidneys, while 20% is secreted directly from the thyroid gland (18, 19). In some diseases of the thyroid gland, the ratio of serum free T_3_ (FT_3_) to free T_4_ (FT_4_) can be altered. In case of inadequate intake of iodine, the T_3_:T_4_ ratio increases, presumably because the synthesis of T_4_ required 25% more iodine compared to T_3_ (20). Altered T_3_:T_4_ ratio was found in rats with iodine shortage (21) and in humans undergoing treatment with levothyroxine (L-T_4_). Arntzenius et al. demonstrated that the autoregulatory response to a lower iodine intake leads to decreased levels of circulating T_4_ (22), while serum T_3_ remains stable, or might even increase (23, 24). Altered T_3_:T_4_ ratios during endocrinological examination might be exploited as a novel tool for identifying iodine insufficiency.

## Iodine

Iodine, a trace element found in soil and seawater, is relatively abundant in seafood, fruit and vegetables. It is ingested in several chemical forms that are reduced to iodide (25), which is absorbed in the stomach and in the duodenum. About 90% is excreted in the urine within 24–48 h (3, 7, 25, 26). Iodine is also released into the mammary gland in the breast milk to provide for the newborn (11, 27). The only physiological role known for iodine is the synthesis of thyroid hormones, comprising 58% of T_3_'s weight, and 65% of T_4_'s (2). Once T_4_ and T_3_ are degraded peripherally, iodide re-enters the plasma iodine pool and can be taken up again by the thyroid gland or excreted by the kidneys (10). When the thyroid works normally, plasma iodine has a half-life of about 10 h, shorter when the thyroid becomes overactive (25). Dietary iodine naturally occurs in the ion form, as potassium or sodium iodide, which is the preeminent substrate of the specific metabolism of thyrocytes, controlling thyroid functions. Either *in vitro* and *in vivo* iodide main roles are: (1) decreasing the response of TSH; (2) inhibiting its own oxidation (the Wolff-Chaikoff effect); (3) reducing its trapping after a delay; (4) at high levels, inhibiting thyroid hormone secretion (28). The Wolff–Chaikoff effect is an autoregulatory phenomenon that prevents the thyroid gland from synthesizing and releasing large quantities of thyroid hormones (19, 29) through the inhibition of organification when inorganic iodide levels in thyrocytes are too high (30, 31). The mechanism responsible for such effect is still unknown, but it may be ascribable to the inhibitory effect of iodide on TPO or other enzymes (19). Indeed, iodide is able to block the cyclic adenosine monophosphate cascade and the Ca^2+^-phosphatidylinositol 4, 5-bisphosphate (PIP2) cascade in thyrocytes and to induce H_2_O_2_ generation (28). H_2_O_2_ is crucial for the TPO-catalyzed thyroid hormone formation (32). In 1993, Chen at al. demonstrated *in vitro* that H_2_O_2_ specifically regulates iodide transport and organification in a dose-dependent manner (33). High H_2_O_2_ concentrations inhibit these functions and can be detrimental to the thyroid, although protection mechanisms prevents damages to the thyrocytes under physiological conditions (34). Intrathyroidal H_2_O_2_ generation, first reported about 50 years ago (35), and iodination are both stimulated by TSH. H_2_O_2_ can regulate iodination either directly, as a substrate of TPO, or indirectly by regulating the activity of TPO when iodide and Tg concentrations are kept constant.

## Myo-Inositol

Myo-inositol, a carbocyclic polyol, belongs to the inositol family. Of nine possible structural isomers, it is the most widely distributed in nature, being present in fresh fruits, vegetables, cereals, legumes, and nuts. Myo-inositol is a fundamental component of structural lipids in cell membranes (36), such as phosphatidylinositol (PI) and the various phosphatidylinositol phosphates (PIPs). Myo-inositol is endogenously synthesized from glucose-6-phosphate and represents, in some tissues, about 99% of intracellular inositol. Initially incorporated at the level of cell membranes as phosphatidyl-myo-inositol, it is a precursor for many inositol-containing compounds involved in membrane biogenesis, signal transduction, vesicle trafficking and chromatin remodeling. It exerts important physiological functions, such as cell and tissue development, lipid synthesis, cell growth, and metabolism regulation. Myo-inositol is one of the precursors for the synthesis of PIPs, which are a source of second messengers like diacylglycerol. The latter regulates numerous species: members of the protein kinase C family; phosphatidylinositol-3,4,5-phosphate; inositol-1,4,5-triphosphate, which modulates intracellular calcium levels. As precursor also of the second messenger phosphoinositide, myo-inositol is involved in cell signaling and regulates the activities of hormones such as TSH, follicle-stimulating hormone (FSH) and insulin (37–39). It recently proved to be a very efficacious and safe treatment for subclinical hypothyroid patients with Autoimmune Thyroiditis (40–46). In 2013, Nordio and Pajalich demonstrated that treatment with myo-inositol plus selenium for 6 months in patients with subclinical hypothyroidism reduced significantly TSH concentrations by 31% (4.4 ± 0.9 mIU/mL vs. 3.1 ± 0.6 mIU/mL), compared to the control group treated only with selenium. These results were later corroborated by the same authors in a further clinical trial. Another study monitored TSH levels in Hashimoto patients with subclinical hypothyroidism receiving myo-inositol plus selenium for 6 months: significant decrease after only 3 months of treatment (from 4.38 ± 0.31 mIU/L at baseline to 3.42 ± 0.3 mIU/L) was observed, and even a greater reduction after 6 months (4.38 ± 0.31 mIU/L at baseline to 3.11 ± 0.2 mIU/L) was reported. No significant changes in the TSH levels in the control group, treated with only selenium, were observed (45). These data demonstrated that myo-inositol plus selenium effectively reduce TSH levels after a short-term supplementation, with even better results achieved after longer periods of treatment. Ferrari et al. confirmed that TSH levels significantly decreased in Hashimoto patients with subclinical hypothyroidism receiving myo-inositol plus selenium for 6 months. Interestingly, among these patients 46% of cases reached the normal TSH range (46). Furthermore, the treatment improved the levels of hormones and antibodies. In different studies, the action of myo-inositol was reported to balance the normal production of T_3_ and T_4_ hormones in patients with autoimmune thyroiditis (40, 43–45), as well as to reduce the blood TPO and Tg antibodies (47). A clinical trial reported a reduction in the antibody titer of 44 and 48%, respectively for anti-TPO and anti-Tg (40). A very recent study confirmed the immunomodulatory effect of myo-inositol combined with selenium in patients with autoimmune thyroiditis. In particular, the treatment maintained the euthyroid state, reducing the risk of developing either subclinical or over hypothyroidism (42).

**Figure 1:**
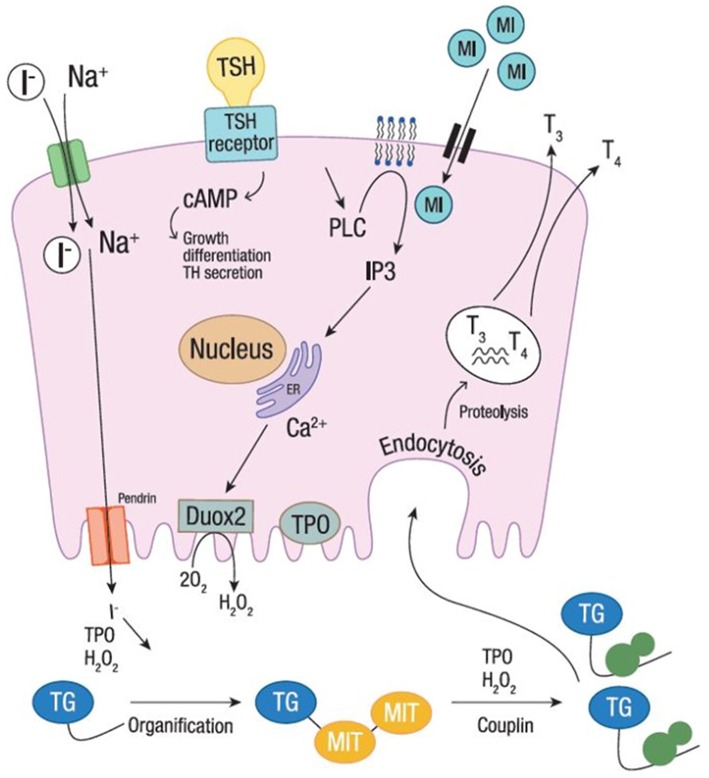
Iodine organification. Iodine organification into thyroid follicular cells. Iodide uptake is mediated by the sodium-iodide symporter, through the gradient generated by Na^+^. TPO, using H_2_O_2_ produced by DUOX2 system, mediates the oxidation, organification and coupling. The secretion of thyroid hormones is shown at the basolateral membrane. Myo-inositol regulates H_2_O_2_-mediated iodination through the phospholipase C-dependent inositol phosphate Ca^2+^/diacylglycerol pathway, resulting in a boost of H_2_O_2_ generation. The cAMP cascade, induced by the TSH activity (through the TSH receptor activation), is also shown. I^−^, Iodine; MI, Myo-inositol; TSH, thyroid stimulating hormone; T_3_, triiodothyronine; T_4_, thyroxine; TPO, thyroid peroxidase; Tg, thyroglobulin; H_2_O_2_, hydrogen peroxide; MIT, monoiodotyrosine; TH, thyroid hormone.

The studies evaluating fT_3_ and fT_4_ reported a significant increase of serum fT_4_ levels after 6-month treatment with myo-inositol plus selenium, although remaining in the normal reference range (43, 45). Both studies reported significantly higher fT_4_ in patients treated for 6 months (from 1.05 ± 0.02 ng/dL at baseline to 1.142 ± 0.03 ng/dL, and from 0.93 ± 0.15 ng/dL at baseline to 1.06 ± 0.14 ng/dL, respectively). Interestingly, myo-inositol helps thyroid-hormone-producing cells to become more efficient and faster at building T_4_ (32, 41). This might be ascribable to a higher availability of iodine, whose organification is boosted by myo-inositol action. Indeed, myo-inositol is involved in one of the first steps of thyroid hormone production and modulates the H_2_O_2_-mediated iodination through the phospholipase C-dependent inositol phosphate Ca^2+^/diacylglycerol pathway, resulting in increased H_2_O_2_ generation (Figure 1). Differently, the cAMP cascade, induced by the TSH activity (through the TSH receptor activation), is more involved in cell growth and differentiation, and in thyroid hormones secretion. As myo-inositol plays a crucial role in the regulation of iodine organification, supplementation may promote faster recovery from ID. Indeed, H_2_O_2_ generated under the stimulus of myo-inositol is available for iodine incorporation inside the thyroid (33, 48). Such activity makes myo-inositol very appealing as a novel molecule to increase iodine availability.

## Conclusion

The use of myo-inositol has gained a positive opinion in the endocrinological field for the restoration of proper thyroid functioning. To date, endocrinologists and gynecologists recommend taking myo-inositol for several benefits in different pathologies, from thyroid diseases to polycystic ovary syndrome and gestational diabetes. Due to its action in regulating iodine organification and thyroid hormone biosynthesis, myo-inositol supplementation along with iodine may improve thyroid functionality and possibly lead to a faster recovery from ID. Under normal conditions, the thyroid gland preferentially synthesizes T_4_ whereas, in pathologies such as ID, T_4_ biosynthesis is closer to the lower cut-off limit of normality. When iodine intake is insufficient, the T_3_:T_4_ ratio increases along with the decrease of T_4_ production, which however remains within the reference range. These subtle fluctuations might describe the patient's iodine status, representing a new simple approach to diagnose ID. Under certain circumstances the supplementation of myo-inositol, along with iodine can be used to restore the normal balance of T_3_:T_4_. Clinical studies to investigate the combined effect of myo-inositol and iodine in counteracting ID are warranted. They might open new scenarios and pose novel challenges for clinical experts and researchers to prevent and treat ID.

## Author Contributions

DB and BO wrote the first draft of the manuscript. VU wrote sections of the manuscript. All authors contributed to manuscript revision, read, and approved the submitted version.

### Conflict of Interest Statement

VU and BO are employees at Lo.Li. Pharma, Rome, Italy. The remaining author declares that the research was conducted in the absence of any commercial or financial relationships that could be construed as a potential conflict of interest.
